# Hay Fever and Hay Asthma

**Published:** 1892-06

**Authors:** P. Watson Williams

**Affiliations:** Physician in charge of the Throat Department at the Bristol Royal Infirmary


### HAY FEVER AND HAY ASTHMA.

BY

P. WATSON WILLIAMS, M.D. Lond.,

*Physician in charge of the Throat Department at the Bristol Royal Infirmary.*

---

OUR knowledge of the pathology of hay fever, and of the neuroses that frequently complicate it, has of late years become more exact and scientific, and consequently the treatment of these affections has been placed on a more rational basis with great advantage to the sufferers. Most cases may be partially, and many completely relieved, while a fair proportion may be altogether cured; it is important, therefore, that the varying conditions which may give rise to the symptoms be clearly recognised and differentiated.

Clinically we may arrange paroxysmal sneezers in three classes: (1) Those in whom the lesion is entirely peripheral, such as the earlier and milder forms of true hay fever, in which the symptoms appear only when the patient is exposed to pollen, etc., and for these, local treatment rarely fails; (2) Those in whom the symptoms, in the first place induced by peripheral irritation, have become more or less persistent, even in the absence of any pollen or special irritating particles; (3) Idiopathic cases, not due to peripheral irritation at all. It is with the pathology and treatment of the first two of these divisions that I now propose to deal.

Like most of the writers on the subject, I was myself a sufferer from hay fever, till I adopted a mode of treatment which afforded a most welcome and complete relief.

Attention was first seriously directed to hay fever by Dr. Bostock, in a paper read before the Royal Medical and Chirurgical Society, in 1819, on a "Periodical Affection of the Eyes and Chest," and who, nine years later, published his further observations on what he termed "Summer Catarrh." The symptoms at that time were popularly supposed to be due to emanations from plants, but Bostock endeavoured to prove that they were produced by the solar rays. In 1869 Helmholtz propounded the theory that it was due to the action of vibrios. He believed that these existed in the nasal fossæ at all times, but that they were excited to activity by the action of the sun's heat. Other writers have ascribed it to certain animal and vegetable odours, to common dust, to the gouty diathesis, some occult atmospheric influence, etc.

Mr. C. H. Blackley of Manchester has set at rest all possible doubt as to the common exciting cause of hay fever. His experiments have shown that it is due to the action on the mucous membrane of the pollen grains of the following natural orders of plants: Ranunculaceæ, Caryophyllaceæ, Compositæ, Geraniaceæ, Leguminosæ, Graminaceæ, Papaveraceæ, Fumariaceæ, Cruciferæ, Violaceæ, and others. The pollen was applied to the mucous membrane of (1) the nose; (2) the larynx, trachea, and bronchial tubes by inhalation; (3) the conjunctivæ; (4) the tongue, lips, and fauces. In every instance the characteristic symptoms were produced, but the most potent pollen was that of the Graminaceæ. The Anthoxanthum odoratum is generally supposed to be especially active in this respect, but the rye grasses and the pollen of the meadow grass (Poa) and meadow fox-tail, and of barley, wheat and oats, seem to be equally to blame.

The observations of Blackley were subsequently confirmed by Sir Morell Mackenzie, who moreover found, from experiments on persons not subject to hay fever, that pollen is a peculiarly irritating dust. Thus, he found that if pollen from any of the particular grasses referred to be "rubbed into the nasal mucous membrane of a healthy person, a feeling of stuffiness in the nose will come on in the course of an hour or two, and last several hours. The sensation is like that experienced at the commencement of a cold, but there is no sneezing or 'running from the nose.' The effect produced by pollen in the non-susceptible is considerably more active than that of tannin, alum and many substances of much stronger chemical character. Why pollen should prove a much more highly exciting substance to the mucous membrane of some persons than to that of others has not yet been determined." The proclivity is certainly not associated, as Sir Morell suggested, with a special keenness of smell, which implies a nervous apparatus of exceptional sensitiveness; but it is more probably often associated with individual peculiarities in the nostrils.

In America hay fever is known by the name of "Rose-cold;" but in this country, too, roses are sometimes found to give rise to the most severe forms of hay fever. Mackenzie tells us that so common is the scourge in the "Great Republic," that a few years ago a "Hay Fever Association" was founded for the collective investigation of facts and the mutual protection of sufferers.

*Predisposing Causes.*—When the exciting cause of hay fever is universal and practically everyone in this country is exposed to it, while a very small minority are affected, it is obvious that individual predisposition is necessary for the symptoms to appear. It occurs most commonly amongst dwellers in towns and cities, though by no means exclusively. It is an affection which is almost confined to the educated classes, and to those who may be said to be of nervous temperament; but I have seen hay fever in a domestic servant, and an analogous idiosyncrasy to tobacco dust in a factory girl. There is no evidence to show that it is specially associated with the gouty diathesis, as has been suggested, or any other pathological constitution other than a strongly neurotic temperament.

It appears to be more commonly met with in men than in women. Thus in Daly's 81 cases, and in 61 cases seen by Mackenzie, about three in five were men, but my own more limited experience gives the proportion as three males to seven females.

The predisposing "nervous" temperament has a tendency to be hereditary, and therefore, in that sense, the affection is often hereditary.

Hot weather increases the severity of the symptoms, partly, no doubt, from its promoting the formation and liberation of pollen, but also from the fact that the sun's rays alone may cause sneezing by stimulation of the facial and conjunctival branches of the fifth nerve reflexly irritating the nasal mucosæ. The effect of heat, however, is much exaggerated from the fact that the exciting cause prevails during the warm summer months—heat alone cannot cause hay fever. The effect of dust is also merely to increase the irritability of the mucous membrane.

*Local Conditions.*—It is only within the last few years that the importance of pathological conditions of the nasal passages as the essential cause of hay fever has been fully appreciated. I have already alluded to the obvious fact that individual predisposition is essential to the disease, and that it is found in those of the nervous temperament. But of "neurotics" only very few are subject to hay fever, and it is observed, though less commonly, in those who are the reverse of neurotic. We have seen that it has been generally referred to an idiosyncrasy which makes the patient remarkably susceptible to this particular form of irritation. The peculiar susceptibility of some individuals to rhus poisoning has been cited as an instance of such idiosyncrasy, and of this the case reported by Mr. A. W. Prichard [1] affords an excellent example. Another case in point is that of a girl who worked in Messrs. W. D. and H. O. Wills' tobacco factory. She complained of incessant sneezing, which came on as soon as she entered the workroom, and continued till she went out. She was very anxious to work, and at first managed to do so, in spite of her sneezing to such an extent that she was nicknamed "The Sneezer," an appellation which showed her statement to be correct when she told me that her case was unique there. So incessant did her sneezing become in the course of a month or so, that if she went to her work she had to come away very soon. As she never suffered from hay fever or paroxysmal sneezing outside that factory, this may be taken as a clear case of idiosyncrasy to tobacco dust, and it is only reasonable to allow that an analogous idiosyncrasy must be held to account for some cases of hay fever and hay asthma.

But the fact that local pathological conditions in the nasal passages are frequently the real cause of the disease

[1] *Bristol Med.-Chir. Journ*, vol. ix., 1891, p. 22.

is equally placed beyond doubt by the many radical cures effected by local treatment. The more attention is paid to the nose the greater is the proportion of cures, and the great importance of recognising these curable cases can hardly be exaggerated.

Ten years ago Dr. Roe of New York reported the excellent results he had obtained from the treatment of the hypertrophied erectile tissue of the nose, which has been observed in every instance in those who are the subjects of hay fever. He has directed attention to the spurs, the spiculæ of bone which are often found projecting into the nasal passages from the turbinated bones and from the septum, and which set up irritation in their neighbourhood, causing more or less chronic rhinitis or nasal catarrh at some other period of the year, although often not sufficiently severe to give rise to much annoyance. It is to the observations of Dr. Roe that the credit is due for the recognition of those local conditions as the cause of the disease, and in the main his remarks have been amply confirmed by many laryngologists.

The abnormalities that are met with in hay-fever subjects are: (1) Those known generally by the term hypertrophic rhinitis, in which the mucosa of the floor and of the septum, but especially the tissue covering the inferior and middle turbinated bones, is thickened or unduly vascular; (2) Spurs and bony projections of the turbinated bones; (3) Deviations of the septum, causing partial or complete occlusion of one nasal passage; (4) Polypi and adenoid hypertrophy of the naso-pharynx; (5) Peculiarly sensitive areas in the lower half of the septum and the floor of the nasal fossæ. Chronic pharyngitis with relaxed fauces and elongated uvula are frequently set up by repeated attacks, and more or less persistent asthma and consequent bronchitis are not unusual in the graver cases.

But those who espouse the purely anatomical lesion theory are probably as much in error as those who adhere to the old idea that the symptoms are wholly attributable to pollen. For the symptoms to occur, three factors are generally necessary: (1) The predisposing constitutional condition; (2) An external irritant, viz., pollen of grasses, etc. in hay fever, of roses in rose fever; (The rare instances of asthma induced by the presence of dogs or cats or horses, are analogous cases.) (3) A pathological condition in the nasal passages, forming or causing a sensitive area.

*The Symptoms* in the majority of cases begin in Great Britain in the first week in June, recurring each year with an almost ludicrous punctuality on about the same date and lasting to the middle of August. In America the first onset is generally about the middle of August. At first only a slight itching of the inner canthus is observed, and watering of the eyes. In a day or two some irritation in the nose with watery discharge comes on, and pricking and dryness in the throat. Very soon attacks of sneezing supervene, and recur with increasing frequency, without the pleasant sense of relief that is usually associated with a good sneeze. The conjunctivæ become injected, the eyes bloodshot, and the nasal passages more or less blocked up by the swelling of the mucous membrane and turgid erectile tissues. The soft palate and uvula become relaxed, and all the painful symptoms of "clergyman's sore throat" are experienced, even ordinary conversation being an effort.

At first the symptoms are merely annoying, but with each annual recurrence they become more and more severe, and life is rendered a perfect misery during the three brightest months of the year. The symptoms are associated with intense prostration, altogether out of proportion to the local irritation, and health is greatly impaired for many weeks after the peculiar symptoms have subsided.

In the worst cases attacks of spasmodic asthma come on early, and sometimes the patient is never free from asthma for weeks continuously. The asthmatic condition persists for some time after the hay fever has disappeared, and the period of persistence tends every year to lengthen, till the unhappy sufferer becomes a confirmed asthmatic, and is never free from the attacks throughout the year. A patient recently under my care had suffered from spasmodic asthma since early infancy, and he could not remember any time in his life when he was not subject to attacks. Unlike most asthmatics, he was worse in summer, and I have no doubt from his description that he suffers from true hay fever in the summer months and that it was the original cause of his asthma. He is an example of a large class of asthmatics, the source of whose trouble is the sensitive area in the nose, stimulation of which causes reflexly contraction of the smaller bronchial tubes. Sir Andrew Clarke is of opinion that hay asthma is due to the sudden swelling of the mucous membrane of the bronchial tubes, causing partial occlusion of the lumen. Blackley's observations on the result of inhaling pollen lend some support to this view; but it is difficult to understand why a non-erectile tissue should undergo sudden swelling, and there is hardly sufficient evidence to justify the theory that hay asthma is produced otherwise than by reflex contraction of the unstriped muscular fibres of the smaller bronchi. Nevertheless there is almost invariably bronchial irritation of greatly varying intensity, with thin watery mucous or muco-purulent expectoration.

Until recently the *treatment* of the affection under consideration has been, as Mr. Lennox Browne so aptly puts it, not only most irrational, but (if one may use such a term) cowardly: for instead of attacking the predisposing idiosyncrasy, or the local hyperæmia, the former has been accepted as inevitable, and the latter ignored; while as to the exciting irritant agent, it has simply been shut out. Of course for wealthy patients with ample leisure, nothing is pleasanter and more effectual than a sea voyage, removal to a "hay fever resort," or residence at the seaside during the hay fever period, but the majority of sufferers are not so happily circumstanced as to render such measures possible.

With the object of excluding the pollen-grains, patients are often advised to plug the nostrils with medicated wool, and to wear goggles, and in the case of women to wear a thin gauze veil when out of doors. Such methods are very ineffectual, and anyone who has tried plugging the nostrils with wool in summer will hesitate before suggesting it to a patient.

During the paroxysms of hay asthma palliative treatment alone avails, but a great deal may be done towards warding off the attacks by the administration of strychnine and arsenic in *small* doses. Sedatives, unfortunately, always leave the patient worse for the remedy, even if it has been necessary for temporary relief; and when, as is often the case in asthma, their frequent repetition is called for, the amount of harm they do is no light matter. "In order to form a conception of the manner in which strychnine relieves asthma, it must be borne in mind that under no circumstances does spasm exist in virtue of any undue supply of nerve-tone to the bronchial muscles, but rather to a perverted or depressed state of the pulmonary nerve supply, both central and peripheral, and a consequent loss of respiratory co-ordination. It is reasonable to believe, therefore, that any agent like stramonium, tobacco, lobelia, nitro-glycerin, pilocarpine, morphine, etc., which possesses the power of narcotizing or paralyzing the nerves which incite the bronchial spasm may also relieve the grip of the latter; but it is also clear that the nerves whose integrity is already impaired are enfeebled still further by such a procedure,—which at best has but a temporary influence. The most rational plan of treatment is one which elevates the tone and increases the normal resistance of the pulmonary nerves." [1]

The importance of treating the constitutional condition by promoting the health and strength in any hay fever subject is very generally recognised, and the administration of tonics, such as iron, quinine, and arsenic, a course of massage, the regulation of the bowels, etc., for a few weeks before the usual period of onset of the characteristic symptoms, will do much towards lessening the severity of the attacks and preventing the general impairment of health which they induce.

Local treatment may be divided into two classes: (*a*) radical, (*b*) palliative.

Radical treatment generally gives very excellent results in simple hay fever, and not infrequently a perfect cure is obtained in cases attended with asthma. Every case should be carefully examined for any of the abnormalities in the nasal passages previously alluded to. On examining first without using cocaine, a thickened vascular condition of the mucous membrane of the inferior turbinated bone and other structures is very often found, and by passing a probe in various directions, sensitive areas

[1] T. J. Mays, *Internat. Clinics*, vol. ii., p. 63

may generally be detected. By spraying the nostrils with a solution of cocaine the vascular swelling of the mucous membrane may be made to subside, and the existence of any spur or spicula projecting from the turbinated bones, or of any deviation in the septum, becomes obvious if present.

If only thickening of the mucosa and sensitive areas is present, a method of treatment I have employed, and which in several cases has been completely successful, consists in spraying the nose with a solution of "iodic-hydrarg." (1 in 1000).[1] As soon as the effect of the cocaine passes off, the pain is intense and lasts for two hours, and therefore I generally give morphine hypodermically immediately after the spray has been used. The mucous membrane of the nose rapidly swells till the nose becomes blocked. The conjunctiva becomes suffused and considerably irritated. In about an hour and a half or two hours the swelling and pain subside, with a condition similar to common nasal catarrh lasting for twenty-four or thirty-six hours. In suitable cases if this be efficiently done at the onset of the symptoms of hay fever, the patient will remain free throughout the season, and there are very few persons who have endured the annoyance and depression of health that hay fever produces who will not gladly undergo this or any treatment which promises relief. This method has the advantage of leaving the sense of smell unimpaired and involves no destruction of tissue, but it differs in detail only from the procedures recommended by many others. Thus Sir Andrew Clarke found great benefit in about half his cases from painting the nose with a mixture of glycerine of carbolic acid, quinine, and perchloride of mercury. Sir Morell Mackenzie had

[1] This is a soluble preparation of iodide of mercury, prepared by Messrs. Burroughs, Wellcome & Co.

no faith in Sir Andrew Clarke's method. He had not tried it himself, but had seen three cases in which carbolic acid was employed by others in hay fever, and in none of them did it produce any permanently satisfactory results. "With regard to the other drugs suggested by Sir Andrew Clarke, it has been abundantly proved that quinine is perfectly useless in hay fever; and as there is nothing of a septic nature in the disease, it is difficult to understand what can be the advantage of applying either that or perchloride of mercury."

On the other hand, Dr. Greville Macdonald has seen good results from spraying with very weak chromic acid solution. These applications owe their efficiency to their irritating character, and not to any antiseptic property. The use of quinine as suggested by Helmholtz is useless, and is condemned by nearly all observers who have tried it.

Hyperplasiæ of the mucosa, and especially thickening over the inferior turbinated, should be reduced by the galvano-cautery, and any sensitive spots should also be cauterised. The senseless destruction of large areas of the mucous membrane is to be deprecated as worse than useless. The hand should always be guided by the eye and the hyperæsthetic areas only be cauterised. Bony spiculæ should be removed by scissors, drill, or gouge. A deviated septum should be straightened, or bony prominences rapidly removed by a dentist's drill. Polypi, if found, must be snared and treated in the proper manner.

Many a case of hay fever, of hay asthma, and also of ordinary spasmodic asthma, will be cured by the treatment of these deformities. Some of the apparently most hopeless cases give the best results. On the other hand, a certain proportion of failures will inevitably occur; but when everything else has been tried, we shall not have done justice to a patient till the abnormalities, if present, have been removed.

All local *palliative measures* are eminently unsatisfactory; for though effectual at first, they soon lose their power, or at any rate their action is but temporary and requires constant repetition. Their number is legion. Ferrier's snuff and menthol snuffs are sometimes of service. Cocaine in any form whatever is to be condemned. Its good effect is very temporary, and its bad effects both local and general are not long in appearing. Hinket of New York advocates the use of a 4 per cent. solution of antipyrin instead of cocaine. For inhalation the vapor benzoini and v. benzoini c. chloroform. of the Throat Hospital Pharmacopœia are very soothing, as also are terebene, pinol, eucalyptus, creasote, or camphor inhalations. Nitre papers, made by soaking six thicknesses of blotting-paper in a saturated solution of nitrate or chlorate of potash, and, after drying, sprinkled with spirits of camphor, tinct. benzoini co., tinct. sumbul, or stramonium burned and the vapour inhaled, also are useful.

It is a good plan to paint the inner canthus and palpebral conjunctiva with a weak solution of perchloride of mercury or nitrate of silver, commencing some days before the symptoms appear. The beneficial effect is due to their rendering the mucous membrane less susceptible, and not to their antiseptic or germicidal properties.

Dr. Gaul reports great success from the employment of a decoction of bidens bipinnata (Spanish needle), a popular remedy in the United States for the cure of asthma.

Hypodermic injections of morphine, or a combination of the fluid extracts of quebracho, grindelia robusta, ethereal tincture of lobelia, given frequently in hot water and brandy, will give rapid relief in a paroxysm of hay asthma.

